# Modeling and Analysis of Upright Piezoelectric Energy Harvester under Aerodynamic Vortex-induced Vibration

**DOI:** 10.3390/mi9120667

**Published:** 2018-12-17

**Authors:** Jinda Jia, Xiaobiao Shan, Deepesh Upadrashta, Tao Xie, Yaowen Yang, Rujun Song

**Affiliations:** 1 School of Mechatronics Engineering, Harbin Institute of Technology, Harbin 150001, China; jiajinda@hit.edu.cn (J.J.); shanxiaobiao@hit.edu.cn (X.S.); 2 School of Civil and Environmental Engineering, Nanyang Technological University, 639798, Singapore; upadrashta@ntu.edu.sg; 3 School of Mechanical Engineering, Shandong University of Technology, Zibo 255049, China; songrujunok@126.com

**Keywords:** energy harvesting, aerodynamics, vortex-induced vibration, distributed modeling, nonlinear analysis

## Abstract

This paper presents an upright piezoelectric energy harvester (UPEH) with cylinder extension along its longitudinal direction. The UPEH can generate energy from low-speed wind by bending deformation produced by vortex-induced vibrations (VIVs). The UPEH has the advantages of less working space and ease of setting up an array over conventional vortex-induced vibration harvesters. The nonlinear distributed modeling method is established based on Euler–Bernoulli beam theory and aerodynamic vortex-induced force of the cylinder is obtained by the van der Pol wake oscillator theory. The fluid–solid–electricity governing coupled equations are derived using Lagrange’s equation and solved through Galerkin discretization. The effect of cylinder gravity on the dynamic characteristics of the UPEH is also considered using the energy method. The influences of substrate dimension, piezoelectric dimension, the mass of cylinder extension, and electrical load resistance on the output performance of harvester are studied using the theoretical model. Experiments were carried out and the results were in good agreement with the numerical results. The results showed that a UPEH configuration achieves the maximum power of 635.04 μW at optimum resistance of 250 kΩ when tested at a wind speed of 4.20 m/s. The theoretical results show that the UPEH can get better energy harvesting output performance with a lighter tip mass of cylinder, and thicker and shorter substrate in its synchronization working region. This work will provide the theoretical guidance for studying the array of multiple upright energy harvesters.

## 1. Introduction

With rapid development technologies, such as portable electronic devices, micro electro mechanical systems (MEMS) and wireless sensors, the reliance on the traditional battery has become a major obstacle due to expensive replacement, bulky volume, limited lifetime, and time-consuming maintenance. One reasonable way to overcome these problems is by converting ambient vibrations or aerodynamic oscillations into useful electrical energy through effective energy harvesters [1,2,3,4,5]. The procedure of harvesting energy can be achieved by different mechanisms including electrostatic [6,7], electromagnetic [8,9,10,11], and piezoelectric transductions [12,13,14,15]. Among these mechanisms, the piezoelectric mechanism attracts more attention due to the high-density output power and simple configuration of energy harvesters. Piezoelectric energy harvesters are widely applied in self-energizing devices or implanted devices [16,17,18], wireless sensors, civil structural monitoring [19], and medical health examination [20,21]. Considerable research efforts have been devoted to energy collecting under base excitations [22,23,24]. The major problem of these energy harvesters is that the output power drops considerably when the frequency of excitation resource varies slightly from the natural frequency of the energy harvester.

In addition to these mechanical vibrations, there have been some researches focused on harvesting potential energy from vibrations induced by environmental fluids. When a supported structure is subjected to air fluid, aeroelastic instability can cause a large-amplitude limited-cyclic oscillation, and such vibration energy can further be converted into usable electricity through the piezoelectric effect. In accordance with the relationship between vibration amplitude and velocity, fluid-induced vibration can be classified as wake-induced [25,26], vortex-induced and flutter-induced vibration [27] and galloping vibration [28,29]. When the fluid flows through a non-streamlined structure (such as a cylinder), the vortex shedding alternately generated at the free end of the structure causes periodic pulsating feedback pressure at both ends of the structure to generate periodic non-linear transverse vibration. Vortex-induced resonance occurs when the frequency of vortex shedding is close to the natural frequency of structure (lock-in). Akaydin et al. [30] investigated a self-excited piezoelectric energy harvester consisting of a cylinder attachment, and the experimental output power was approximately 0.1 mW of non-rectified electrical power at a flow speed of 1.192 m/s. Weinstein et al. [31] put a cylinder bluff ahead of the cantilever to cause vortex-induced vibrations. Zero point two milliwatts and 3 mW output energies were obtained at the wind speed of 2.5 m/s and 3 m/s, respectively. Mehmood et al. [32] studied numerical simulations on a series of Reynolds numbers covering pre-synchronization, synchronization, and post-synchronization areas using the linear mathematical model and the effect of the load resistance oscillation amplitude, lift coefficient, voltage output, and harvested power was obtained. Gao et al. [33] tested upright piezoelectric energy harvester (UPEH) in laminar flow generated by a wind tunnel and in turbulent flow generated by the fan. The result data showed that the UPEH obtained a higher voltage in turbulent flow than in laminar flow and the turbulence excitation is the dominant mechanism under vortex-induced vibrations (VIVs) in the lock-in region. Dai et al. [34] designed and tested four distinct harvester configurations consisting of the same dimensions of the piezoelectric beam and the same cylinder extension. The results showed that the T-shape piezoelectric energy harvester (TPEH) whose cylinder was perpendicular to the beam should be operated at higher wind speed while the UPEH can obtain more energy at low speeds. As for modeling theory, Barrero–Gil et al. [35] presented a one-degree-of-freedom model. The cylinder was supported with a spring and damper while undergoing VIVs. The fluid forces were obtained by experimental vibration tests. Facchinetti et al. [36] modeled the near wake dynamics to describe the motion of the bluff cylinder by a classical van der Pol equation. Dai et al. [37] derived the distributed-parameter theory of TPEH. An approximate mode function was used to describe the efficiency of TPEH. 

However, to better use the working area, a set of energy harvesters will be used simultaneously for generating more energy in practice. The characteristic of a large working space of the TPEH may be a drawback for multiple energy harvester arrays and, thus, lower the total electricity energy output. Compared to a TPEH, the best advantage of the UPEH is that it takes less working space, so it is more suitable for two or more tandem energy harvesters working together to generate more energy. This will be a research hotspot of energy harvesting in the future.

Various mathematical modeling methods for piezoelectric energy harvesters have been proposed. Williams [38] proposed the uncoupled single-degree-of-freedom model. The energy harvester was simply equivalent to the mass–spring–damping system. The dynamic vibration equation was solved to obtain the stress and strain of the piezoelectric layer. The output power was calculated according to piezoelectricity, and the influence of output power on system vibration is ignored. Dutoit et al. [39] deduced the single-degree-of-freedom coupled vibration equation and introduced the electric effect of piezoelectricity into the equation. Enturk et al. [40] proposed the configuration of piezoelectric energy harvester with tip mass at the free end. Because the mass of the end mass block was not much more than the mass of the piezoelectric beam, the modified coupling equation was put forward. For more accurate modeling analysis, Scholars proposed the method of distributed parameter modeling. To evaluate the influence of the high-order vibration mode on energy harvester, Erturk [41] established the distributed nonlinear dynamic equation based on the massless piezoelectric beam. However, this kind of modeling method only considers the transverse displacement of the piezoelectric beam and has no regard for the influence of the axial force (pre-tension, vertical gravity) of the cantilever beam. Therefore, to solve this problem, an energy approach is used to model and study the effects of the attached cylinder on the axial pressure of the cantilever beam.

This work aims to derive a detailed model of a UPEH with vortex-induced excitation. In Section 2, a governing coupled mathematical formulation of the harvesting system is developed. Based on Garlerkin discretization, a reduced order is derived. In Section 3, experiments are performed to verify the correction of the theoretical model. In Section 4, the effects of dimensions of substrate and mass of cylinder are shown. The conclusions are provided in Section 5.

## 2. Physics Statement and Mathematical Model

**Figure 1:**
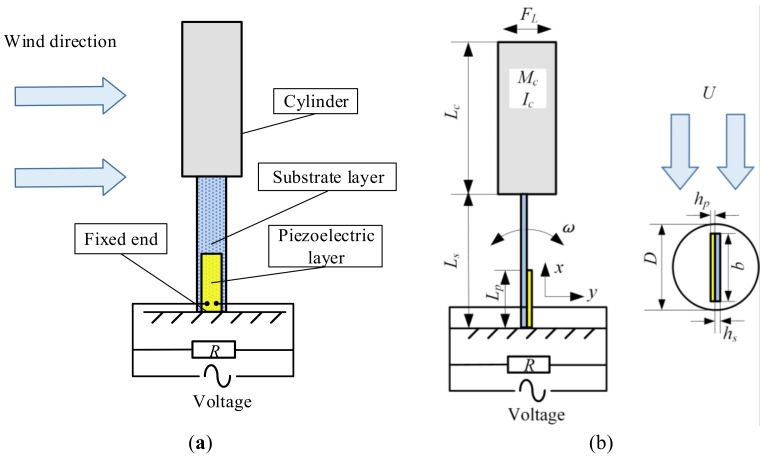
Upright piezoelectric energy harvester (UPEH) energy harvesting system (**a**) composition of UPEH system; (**b**) schematic of UPEH.

Figure 1a shows the composition of UPEH energy harvesting system. The UPEH comprises an upright piezoelectric beam with cylinder extension. The axis of the beam and the axis of the cylinder are in a straight line. The load resistance is attached to the piezoelectric layer by two electrodes. Figure 1b shows the detailed schematic of UPEH. To model UPEH energy harvesting system, the following assumptions are adopted:

(1) The substrate layer and the piezoelectric layer are assumed to be bonded perfectly.
(2) The beam is a Euler–Bernoulli beam. The thickness of beam is supposed to be much less than its length or width. The axial deformation and shear deformation of beam are neglectable.
(3) The cylinder extension is rigid, and its deformation is ignored.
(4) The attachments of the tip cylinder, beam, and unfixed end are assumed to be tight.
(5) The airflow is laminar flow.

In this section, a nonlinear distributed-parameter model of the UPEH was developed using Lagrange’s equations. We first calculate the total kinetic energy *T*, the total potential energy *V* and virtual work *W*. The kinetic energy *T* of the system consists of the kinetic energy of the piezoelectric layer, substrate layer and the cylinder with surrounding flowing air. *T* can be expressed as

(1) $$\begin{array}{ll} T= & \frac{1}{2}\int_{V_{s}}\rho_{s}{\left[\frac{\partial w\left(x,t\right)}{\partial t}\right]}^{2}dV_{s}+\frac{1}{2}\int_{V_{p}}\rho_{p}{\left[\frac{\partial w\left(x,t\right)}{\partial t}\right]}^{2}dV_{p}\\ & +\frac{1}{2}\left(M_{c}+M_{f}\right){\left[{\frac{\partial w\left(x,t\right)}{\partial t}|}_{x=L_{s}}+\frac{L_{c}}{2}{\frac{\partial^{2}w\left(x,t\right)}{\partial x\partial t}|}_{x=L_{s}}\right]}^{2}+\frac{1}{2}\left(I_{c}+I_{f}\right){\left[{\frac{\partial^{2}w\left(x,t\right)}{\partial x\partial t}|}_{x=L_{s}}\right]}^{2} \end{array}$$

where *ρ_s_* and *ρ_p_* are the densities of the substrate layer and piezoelectric layer, respectively. *V_s_* and *V_p_* are the volumes of the substrate layer and piezoelectric layer, respectively. *L_c_* is the length of the attached cylinder, and *L_s_* is the length of the substrate layer. *w(x,t)* is the displacement of the cantilever beam in the *y* direction. *x* is the axial coordinate along the beam length, and *t* is time. *M_c_* is the mass of the cylinder and *I_c_* is the moment of the cylinder defined as *I_c_* = *M_c_*(*L_c_/2*)^2^/3. *M_f_* and *I_f_* are fluid-added mass and fluid-added moment, respectively. These two symbols are given by

(2) $$M_{f}=\frac{C_{M}\rho_{f}\pi L_{c}D^{2}}{4}$$

(3) $$I_{f}=\frac{M_{f}L_{c}{}^{2}}{12}$$

where *C_M_* = 1 [36] is fluid-added mass coefficient.

The total potential energy includes elastic potential energy in the substrate layer, elastic potential energy in the piezoelectric layer, and gravitational potential energy. Therefore, it can be expressed as

(4) $$U=\frac{1}{2}\int_{V_{s}}\sigma_{s}\varepsilon_{s}dV_{s}+\frac{1}{2}\int_{V_{p}}\sigma_{p}\varepsilon_{p}dV_{p}-\frac{1}{2}\int_{V_{p}}E_{3}D_{3}dV_{p}-\frac{M_{c}gL_{c}}{4}{\left({\frac{\partial w\left(x,t\right)}{\partial t}|}_{x=L_{s}}\right)}^{2}$$

The *x*-directional stresses in the substrate layer and piezoelectric layer are given by

(5) $$\sigma_{s}=E_{s}\varepsilon_{s}$$

(6) $$\sigma_{p}=E_{p}\varepsilon_{p}-e_{31}E_{3}$$

where *E_s_* and *E_p_* are the Young’s modulus of the substrate layer and the piezoelectric layer, respectively. *e_31_* is piezoelectric stress coefficient. *E_3_* is the electric field in the piezoelectric layer defined as *E_3_* = *V*(*t*)/*h_p_*, where *V*(*t*) is output voltage, and *h_p_* is the thickness of the piezoelectric layer. *D_3_* is the electric displacement given by

(7) $$D_{3}=e_{31}\varepsilon_{p}+\varepsilon_{33}^{}E_{3}$$

where *ε*_33_ is permittivity at constant strain. The strains of two layers based on the neutral layer are given by

(8) $$\varepsilon_{s}=\varepsilon_{p}=-y\frac{\partial^{2}w\left(x,t\right)}{\partial x^{2}}$$

The virtual work *W* [42] includes the virtual work of load resistance *δW_R_* [37], lift force *δW_L_* [43], fluid drag force *δW_cf_*, and mechanical damping *δW_cm_*. *W* can be expressed as

(9) $$\delta W=\delta W_{R}+\delta W_{L}+\delta W_{cf}+\delta W_{cm}$$

(10) $$\delta W_{R}=-V\left(t\right)\delta Q\left(t\right)$$

where *Q(t)* is the quantity of electric charge between the electrodes of the piezoelectric layer.

(11) $$\delta W_{L}=F_{L}\left(t\right)\delta \int_{0}^{L_{c}}\left[{w\left(x,t\right)|}_{x=L_{s}}+L{\frac{\partial w\left(x,t\right)}{\partial x}|}_{x=L_{s}}\right]dL$$

where *F_L_(t)* is the lift force unit length expressed as [36]

(12) $$F_{L}\left(t\right)=\frac{C_{L}\left(t\right)\rho_{f}DU^{2}}{2}$$

where *U* is the mean velocity of wind flow, *C_L_*(*t*) is the vortex lift coefficient. *D* is the diameter of the cylinder, *ρ_f_* is the density of air flow. Governed by the van der Pol equation [36], *q*(*t*) is introduced as *q*(*t*) = 2*C_L_*(*t*)/*C_L0_*, which is used to describe the behavior on the near wake of the cylinder. *C_L0_* is the reference lift coefficient on an unfixed cylinder undergoing vortex shedding.

(13) $$\ddot{q}+\varepsilon \omega_{f}\left(q^{2}-1\right)\dot{q}+\omega_{f}{}^{2}q=\left(\frac{A}{D}\right)\left[{\frac{\partial^{2}w\left(x,t\right)}{\partial t^{2}}|}_{x=L_{s}}+\frac{L_{c}}{2}{\frac{\partial^{3}w\left(x,t\right)}{\partial x\partial t^{2}}|}_{x=L_{s}}\right]$$

(14) $$\omega_{f}=\frac{2\pi S_{t}U}{D}$$

where *ω_f_* is vortex shedding frequency, and *S_t_* is Strouhal number.

The virtual work of fluid-added damping *δW_cf_* [42] is given by

(15) $$\delta W_{cf}=-c_{f}\int_{0}^{L_{c}}\left[{\frac{\partial w\left(x,t\right)}{\partial t}|}_{x=L_{s}}+{L\frac{\partial^{2}w\left(x,t\right)}{\partial x\partial t}|}_{x=L_{s}}\right]\left[{\delta w\left(x,t\right)|}_{x=L_{s}}+L\delta {\frac{\partial w\left(x,t\right)}{\partial x}|}_{x=L_{s}}\right]dL$$

where *c_f_* is fluid drag force per unit length given by

(16) $$c_{f}=\frac{C_{D}\rho_{f}DU}{2}$$

The mechanical damping *δW_cm_* is

(17) $$\delta W_{cm}=-\int_{0}^{L_{s}}c_{m}\frac{\partial w\left(x,t\right)}{\partial t}\delta w\left(x,t\right)dx$$

where *C_D_* is the mean drag coefficient, and *c_m_* is the mean mechanical damping coefficient.

To characterize the response of the energy harvester and effects of different structural parameters on its output performance, the equations of energy harvesting system are discretized by using the Galerkin procedure to obtain the reduced-order model. The transversal displacement *w*(*x*,*t*) is separated into spatial and time variables as

(18) $$w\left(x,t\right)=\sum_{i=1}^{n}\phi_{i}\left(x\right)r_{i}\left(t\right)$$

where *ϕ_i_*(*x*) and *r_i_*(*t*) are the model shape and model coordinate of the cantilever beam, respectively. The mode function is divided into two parts because the piezoelectric layer does not fully cover the substrate layer. The *ϕ_i_*(*x*) can be determined as

(19) $$\begin{array}{l} \phi_{i}\left(x\right)=\phi_{i1}\left(x\right)=A_{1}\sin\lambda_{i1}x+B_{1}\cos\lambda_{i1}x+C_{1}\sinh\lambda_{i1}x+D_{1}\cosh\lambda_{i1}x,0\leq x<L_{p}\\ \phi_{i}\left(x\right)=\phi_{i2}\left(x\right)=A_{2}\sin\lambda_{i2}x+B_{2}\cos\lambda_{i2}x+C_{2}\sinh\lambda_{i2}x+D_{2}\cosh\lambda_{i2}x,L_{p}\leq x\leq L_{s} \end{array}$$

where *A_1_*, *A_2_*, *B_1_*, *B_2_*, *C_1_*, *C_2,_ D_1_*, and *D_2_* are the coefficients related to the boundary conditions and the coefficients of *λ_i1_* and *λ_i1_* are related by

(20) $$\lambda_{1}{}_{i}=\sqrt[{4}]{\frac{\left(\rho_{s}h_{s}+\rho_{p}h_{p}\right)EI_{2}}{\rho_{s}h_{s}EI_{1}}}\lambda_{2}{}_{i}$$

where *EI_1_* = *b*[*E_s_*(*h_b_^3^*– *h_a_^3^*)+*E_p_*(*h_c_^3^*− *h_b_^3^*)]/3 when 0 ≤ *x* < *L_p_* and *EI_2_* = *bE_s_ h_s_^3^*/12 when *L_p_* ≤ *x* < *L_b_*. *h_a_*, *h_b_* and *h_c_* are the positions of the layers defined with respect to the neutral axis as *h_a_* = − *h_0_*, *h_b_* = *h_s_* − *h_0_*, *h_c_* = (*h_s_* + *h_p_*) − *h_0_*, respectively. *h_0_* is given by

(21) $$h_{0}=\frac{\left(h_{s}+h_{p}\right)E_{p}h_{p}}{2\left(E_{p}h_{p}+E_{s}h_{s}\right)}+\frac{h_{s}}{2}$$

The boundary conditions of energy harvesting system are determined as

(22) $$\begin{array}{ll} {w\left(x,t\right)|}_{x=0}=0 & {\frac{\partial w\left(x,t\right)}{\partial t}|}_{x=0}=0\\ EI_{1}{\frac{\partial^{2}w\left(x,t\right)}{\partial x^{2}}|}_{x=L_{P}} & =EI_{2}{\frac{\partial^{2}w\left(x,t\right)}{\partial x^{2}}|}_{x=L_{P}}EI_{1}{\frac{\partial^{3}w\left(x,t\right)}{\partial x^{3}}|}_{x=L_{P}}=EI_{2}{\frac{\partial^{3}w\left(x,t\right)}{\partial x^{3}}|}_{x=L_{P}}\\ EI_{2}{\frac{\partial^{2}w\left(x,t\right)}{\partial x^{2}}|}_{x=L_{s}} & +\left(I_{c}+I_{f}+\left(M_{c}+M_{f}\right){\left(\frac{L_{c}}{2}\right)}^{2}\right){\frac{\partial^{3}w\left(x,t\right)}{\partial x\partial t^{2}}|}_{x=L_{s}}+\left(M_{f}+M_{c}\right){\frac{L_{c}}{2}\frac{\partial^{2}w\left(x,t\right)}{\partial t^{2}}|}_{x=L_{s}}=0\\ EI_{2}{\frac{\partial^{3}w\left(x,t\right)}{\partial x^{3}}|}_{x=L_{s}} & -\left(M_{f}+M_{c}\right){\frac{\partial^{2}w\left(x,t\right)}{\partial t^{2}}|}_{x=L_{s}}-\frac{L_{c}}{2}\left(M_{f}+M_{c}\right){\frac{\partial^{3}w\left(x,t\right)}{\partial x\partial t^{2}}|}_{x=L_{s}}=0 \end{array}$$

By substituting Equation (19) into Equation (22), we can obtain the simplified boundary conditions as

(23) $$\begin{array}{l} \phi_{i1}\left(0\right)=0\;\;\;\;\phi_{i1}{}^{\prime }(0)=0\\ \phi_{i1}\left(L_{p}\right)=\phi_{i2}\left(L_{p}\right)\;\;\;\phi_{i1}{}^{\prime }\left(L_{p}\right)=\phi_{i2}{}^{\prime }\left(L_{p}\right)\\ EI_{1}\phi_{i1}{}^{\prime \prime }\left(L_{p}\right)=EI_{2}\phi_{i2}{}^{\prime \prime }\left(L_{p}\right)\\ EI_{1}\phi_{i1}{}^{\prime \prime \prime }\left(L_{p}\right)=EI_{2}\phi_{i2}{}^{\prime \prime \prime }\left(L_{p}\right)\\ EI_{2}\phi_{i2}{}^{\prime \prime }\left(L_{s}\right)-\omega_{i}{}^{2}\left(I_{c}+I_{f}+\left(M_{c}+M_{f}\right){\left(\frac{L_{c}}{2}\right)}^{2}\right)\phi_{i2}{}^{\prime }\left(L_{s}\right)-\omega_{i}{}^{2}\left(M_{c}+M_{f}\right)\frac{L_{c}}{2}\phi_{i2}\left(L_{c}\right)=0\\ EI_{2}\phi_{i2}{}^{\prime \prime \prime }\left(L_{s}\right)+\omega_{i}{}^{2}\left(M_{c}+M_{f}\right)\phi_{i2}\left(L_{s}\right)+\omega_{i}{}^{2}\left(M_{c}+M_{f}\right)\frac{L_{c}}{2}\phi_{i2}{}^{\prime }\left(L_{s}\right)=0 \end{array}$$

where *ω_i_* is natural frequency of the UPEH without considering the gravity effect in the *i*th mode. The relationship between different mode shapes can be obtained by applying the following equations [44]

(24) $$\begin{array}{ll} \int_{V_{s}}\rho_{s}\phi_{i}\left(x\right)\phi_{j}\left(x\right)dV_{s}+ & \int_{V_{p}}\rho_{p}\phi_{i}\left(x\right)\phi_{j}\left(x\right)dV_{p}+\left(M_{c}+M_{f}\right)\left[\phi_{i}\left(L_{s}\right)+\frac{L_{c}}{2}{\phi^{\prime }}_{i}\left(L_{s}\right)\right]\left[\phi_{j}\left(L_{s}\right)+\frac{L_{c}}{2}{\phi^{\prime }}_{j}\left(L_{s}\right)\right]\\ & +\left(I_{c}+I_{f}\right){\phi^{\prime }}_{i}\left(L_{s}\right){\phi^{\prime }}_{j}\left(L_{s}\right)=\delta_{ij} \end{array}$$

(25) $$\int_{V_{s}}E_{s}y^{2}\phi_{i}{}^{\prime \prime }\left(x\right)\phi_{j}{}^{\prime \prime }\left(x\right)dV_{s}+\int_{V_{p}}E_{p}y^{2}\phi_{i}{}^{\prime \prime }\left(x\right)\phi_{j}{}^{\prime \prime }\left(x\right)dV_{p}=\omega_{i}^{2}\delta_{ij}$$

where *δ_ij_* is the Kronecker delta. The value of *δ_ij_* is 1 if *i* is equal to *j* and equal to 0 otherwise. Mode shapes and natural frequencies can be obtained. Next, the Lagrange’s equations are used to derive the governing equations of the motion of the harvesting system.

(26) $$\begin{array}{l} \frac{\partial }{\partial t}\left(\frac{\partial L}{\partial {\dot{\eta }}_{i}}\right)-\frac{\partial L}{\partial \eta_{i}}=\frac{\delta W}{\delta \eta_{i}}\left(i=1,2,3,...,n\right)\\ \frac{\partial }{\partial t}\left(\frac{\partial L}{\partial \dot{\gamma }}\right)-\frac{\partial L}{\partial \gamma }=\frac{\delta W}{\delta \gamma }=-\frac{V}{R}\left(\dot{\gamma }=V\right) \end{array}$$

where *L* is the Lagrangian defined as *L = T − V*. The reduced-order fluid–solid–electric coupling model of energy harvester can be obtained as

(27) $$\begin{array}{l} {\ddot{r}}_{i}\left(t\right)+\left(2\zeta_{i}\omega_{i}+\eta_{i}\right){\dot{r}}_{i}\left(t\right)+\left(\omega_{i}^{2}+\mu_{i}\right)r_{i}\left(t\right)+\theta_{i}V\left(t\right)=f_{i}(t)\\ C_{P}\frac{dV\left(t\right)}{dt}+\frac{V\left(t\right)}{R}-\sum_{i=1}^{n}\theta_{i}{\dot{r}}_{i}\left(t\right)=0 \end{array}$$

where *ζ_i_* is the damping ratio of energy harvester working at the *i*th mode. *C_p_* is the capacitance of piezoelectric sheet and is calculated by *C_p_ = ε_33_b_p_L_p_/h_p_*. The parameter *f_i_* (t) represents the lift force on the attached cylinder. This term is given by

(28) $$f_{i}(t)=\frac{C_{L0}\rho_{f}DU^{2}}{4}\left(L_{c}\phi_{12}\left(L_{s}\right)+\frac{L_{c}{}^{2}}{2}{\phi^{\prime }}_{12}\left(L_{s}\right)\right)q\left(t\right)$$

As the energy collector is placed vertically, the cylinder extension at the free end can create pressure on the axial direction of the beam, thus, affecting the vibration characteristics of the energy harvester. The coefficient *μ_i_* is used to express the influence of the gravity of the attached cylinder on the natural frequency of harvester and given by [43]

(29) $$\mu_{i}=-\frac{M_{c}gL_{c}}{4}{\phi^{\prime }}_{i2}\left(L_{s}\right)\sum_{j=1}^{i}{\phi^{\prime }}_{j2}\left(L_{s}\right)$$

The coefficient *η_i_* is expressed as

(30) $$\eta_{i}=\sum_{j=1}^{i}c_{f}\int_{0}^{L_{c}}\left[\phi_{j2}(L_{s})+L{\phi^{\prime }}_{j2}(L_{s})\right]\left[\phi_{i2}(L_{s})+L{\phi^{\prime }}_{i2}(L_{s})\right]dL$$

*θ_i_* is electromechanical coupling coefficient given by

(31) $$\theta_{i}=-\frac{e_{31}b_{p}\left(h_{c}^{2}-h_{b}^{2}\right)}{2h_{p}}{\phi^{\prime }}_{i1}\left(L_{p}\right)$$

where *b_p_* is the width of the active piezoelectric sheet. The first order mode (*i* = 1) is used to study the dynamics model of energy harvester. Introducing the following state variables as

(32) $$X=\left[\begin{array}{c} X_{1}\\ X_{2}\\ X_{3}\\ X_{4}\\ X_{5} \end{array}\right]=\left[\begin{array}{c} r\\ \dot{r}\\ V\\ q\\ \dot{q} \end{array}\right]$$

The space state equation of the energy harvesting system is established in the appendix part and expressed as

(33) $$\begin{array}{l} \dot{X}=\\ \left[\begin{array}{c} X_{2}\\ -\left(\omega_{1}{}^{2}+\mu_{1}\right)X_{1}-(2\zeta_{1}\omega_{1}+\eta_{1})X_{2}-\theta_{1}X_{3}+KX_{4}\\ \frac{\theta_{1}}{C_{p}}X_{2}-\frac{1}{C_{p}R}X_{3}\\ X_{5}\\ -\varepsilon \omega_{f}(X_{4}{}^{2}-1)X_{5}-\omega_{f}{}^{2}X_{4}+\frac{A}{D}\left(\phi_{12}\left(L_{s}\right)+\frac{L_{c}}{2}{\phi^{\prime }}_{12}\left(L_{s}\right)\right)\left(KX_{4}-\left(\omega_{1}{}^{2}+\mu_{1}\right)X_{1}-\theta_{1}X_{3}-(2\zeta_{1}\omega_{1}+\eta_{1})X_{2}\right) \end{array}\right] \end{array}$$

where *X_i_* is the space state variable, and *K* is given by

(34) $$K=\frac{C_{L0}\rho_{f}DU^{2}}{4}\left(L_{c}\phi_{12}\left(L_{s}\right)+\frac{L_{c}{}^{2}}{2}{\phi^{\prime }}_{12}\left(L_{s}\right)\right)$$

## 3. Experimental Validation

**Figure 2:**
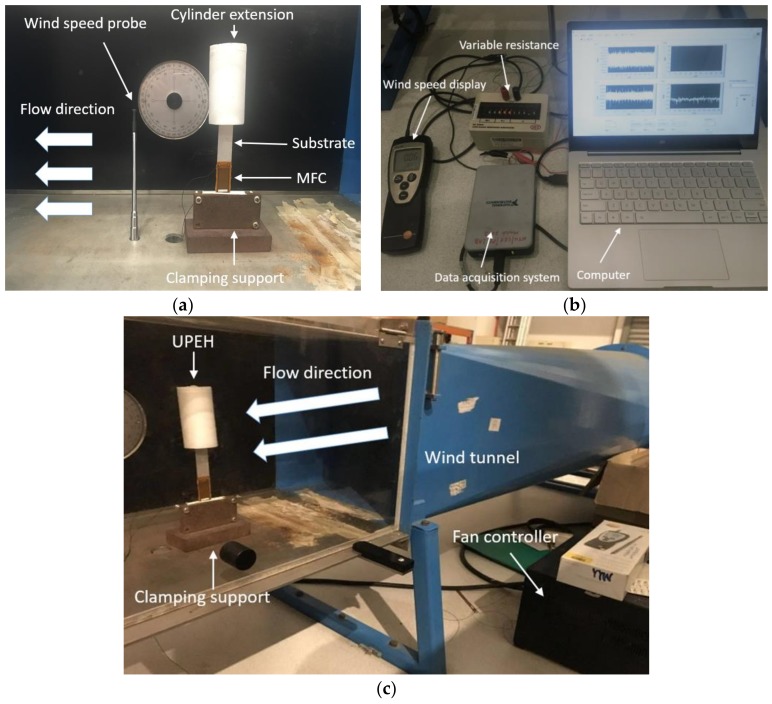
Experimental test platform: (**a**) UPEH setup; (**b**) Power acquisition system; (**c**) Wind tunnel.

Figure 2a shows the experimental setup of a UPEH prototype which is installed in a wind tunnel. The cantilever is fabricated with an aluminum substrate bonded with macro fiber composite (M-2814-P2, Smart Material GmbH, Dresden, Germany) using glue (DP-460, 3M Scotch-Weld, Singapore) from the clamped end. The tip cylinder (Art Friend, Singapore) is made of light foam and bonded with the cantilever by glue. Figure 2b shows the power acquisition system and Figure 2c shows the wind tunnel. The experiments were carried out in the working section of the wind tunnel. The frequency controller of the wind tunnel can change the rotational speed of fans at the end of the wind tunnel, and, thus, change the wind speed. The experiments are carried out at discrete wind speeds. The wind speed is linearly increased/decreased slowly between successive wind speeds, and the transient changing region of wind speed is not taken into consideration in this experiment. The flowing direction of the wind is in the width direction of the cantilever. An adjustable external electrical resistance is used to investigate the influence of resistance on the output performance of energy harvester. The wind speed is measured in real-time by an anemometer (testo 425, Testo SE & Co. KGaA, Lenzkirch, Germany). The voltage across the load resistance is measured by DAQ module (NI 9229, National Instruments, Austin, TX, USA) and processed by LabVIEW software. A computer is utilized to record and store the voltage signal. The average output power on load resistance is calculated by *P=V^2^_RMS_/R* where the RMS voltage is the root mean square voltage. The mechanical damping ratio can be acquired using the logarithmic decrement method by the free vibrating test. The detailed values of the physical properties of the energy harvesting system are listed in Table 1.

**Figure 3:**
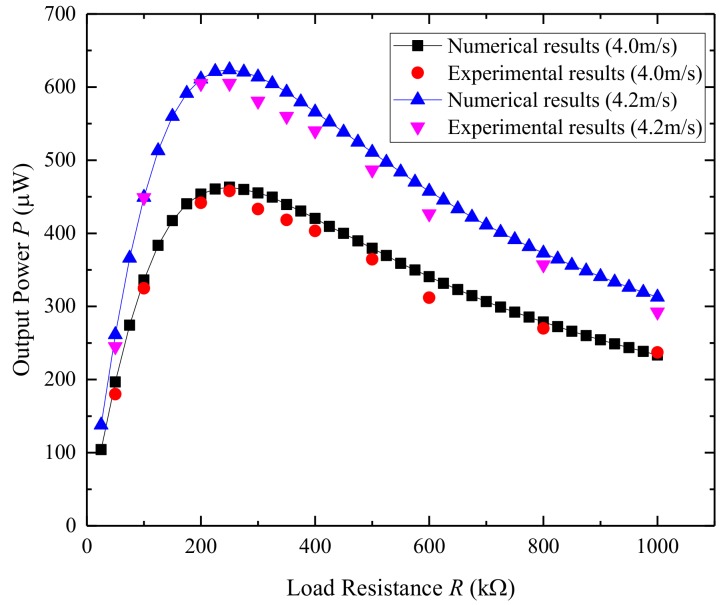
Numerical results and experimental results of average output power *P* versus load resistance *R.*

For investigating the maximum performance of harvesting energy, the load resistance should be taken into consideration. Figure 3 shows the numerical results, and experimental results of the average output power *P* versus load resistance *R*. The powers are measured at a wind speed of 4.0 m/s and 4.2 m/s, respectively. It can be found that with the increase of the load resistance value, the average output power increases first until it reaches the maximum value and then decrease. The value of the resistance when the output power is largest is called the optimum resistance. From Figure 3, we can observe that there is an optimal resistance around 250 kΩ for both wind speeds. The optimum resistance for an energy harvester has little relationship with wind speed. The optimum resistance of experimental results is consistent with that of the numerical analysis.

**Figure 4:**
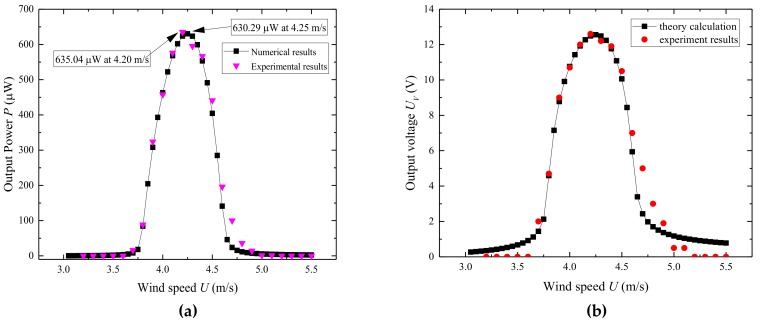
Numerical results and experimental results: (**a**) output power *P* versus wind speed *U;* (**b**) output voltage *U_V_* versus wind speed *U.*

To investigate the effect of wind speed *U*, output powers across the load resistance at various wind speeds were measured and calculated. Both numerical and experimental resistances were set to 250 kΩ, which is assumed to be the optimum resistance for the proposed configuration. Figure 4 shows the average output power and the output voltage versus the wind speed, respectively. From both results, we can observe that with a wind speed increase, output power *P* first increases until it reaches the maximum *P_max_* and then decreases to almost zero. The reason for this result is that there is a positive correlation between the vortex shedding frequency and wind speed. With a wind speed increase, the frequency of vortex shedding increases gradually close to the natural frequency of UPEH. The lift force of vortex pushes the cylinder into the resonance region and, thus enhances the vibration amplitude. As a result, the harvester generates much more power. When the wind velocity exceeds a certain value, the frequency of the vortex shedding exceeds the natural frequency of the cylinder, and little output power can be generated even if the lift force is greater. The maximum average output power *P* is 635.04 µW when the wind speed *U* is 4.2 m/s as obtained in the experiment. The theoretical maximum average output power calculated through the prediction model is 630.29 µW when the wind speed *U* is 4.25 m/s. Meanwhile, the presented theory also predicts the synchronization region well. The theoretical starting and ending wind speeds for energy harvesting are 3.75 m/s and 4.7 m/s, respectively While the starting and ending wind speeds obtained from experimental results were 3.7 m/s and 4.8 m/s, respectively. There is in good agreement between the theoretical and experimental results both in the maximum output energy and the synchronization region. It proves the validity of the theoretical model. 

## 4. Influence of Substrate Dimensions, Piezoelectric Dimensions, and Mass of Cylinder

In this section, the effects of length and thickness of the piezoelectric layer, substrate layer, and tip mass of the cylinder on the natural frequency and output power of the UPEH versus wind speed were studied. It should be mentioned that energy harvesting from VIV only occurs in a definite region of wind velocity when the shedding frequency is near the natural frequency of the harvesting system. Therefore, the synchronization region should be considered in the design phase to meet diverse environmental requirements. For convenience, when one parameter is varied, the other parameters remain the same value. The load resistance *R* is assumed to be 250 kΩ in all the following theoretical simulation.

**Figure 5:**
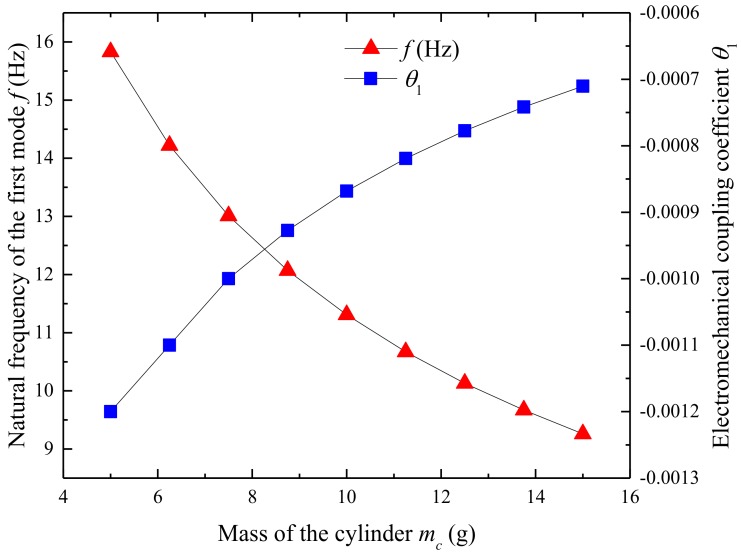
Influence of cylinder mass on the first mode frequency and electromechanical coupling coefficient.

**Figure 6:**
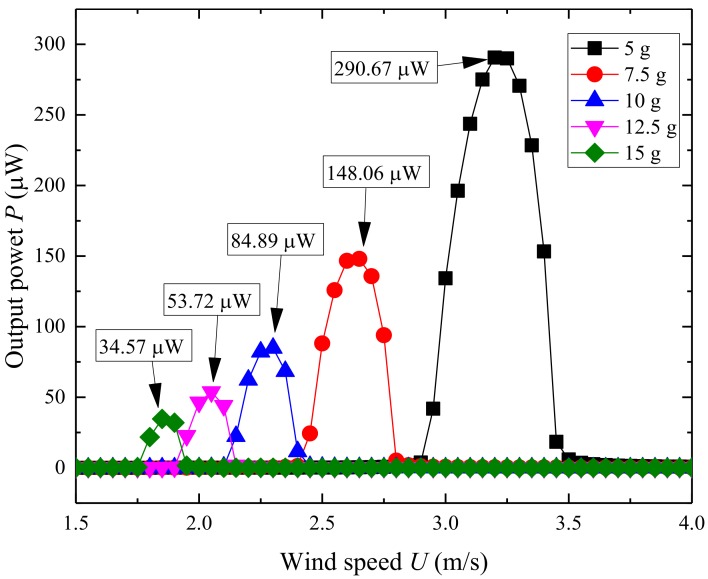
Output power of UPEH versus wind speed with different mass of cylinder.

Figure 5 shows the effect of tip mass on the natural frequency and electromechanical coupling coefficient of UPEH. With the tip mass increasing from 5 g to 15 g, the natural frequency of the energy harvester gradually decreases from 15.83 Hz to 9.27 Hz. And,, hence the resonant vortex shedding frequency decreases and, thus, lower resonant wind velocity. The electromechanical coupling coefficient changes from −0.0012 to −0.0007. This result reveals that the higher mass of the cylinder results in lower electromechanical energy conversion efficiency. Figure 6 illustrates the output power of the UPEH versus wind speed under different masses of the cylinder. From Figure 6, it can be observed that the optimum wind speed becomes lower with an increase in cylinder mass and the maximum output power becomes smaller. The conclusion can be drawn that the synchronization region becomes wider as the cylinder becomes lighter. Although a lighter cylinder gives a high output power and a wider synchronization region, the design of the UPEH is still based on the environmental wind speed.

**Figure 7:**
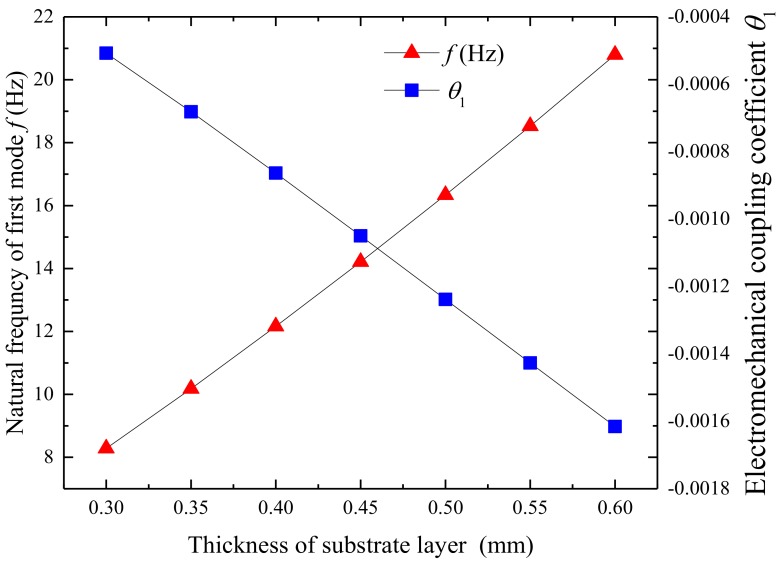
Variation of the first natural frequency and electromechanical coupling coefficient of UPEH as a function of the thickness of substrate layer.

**Figure 8:**
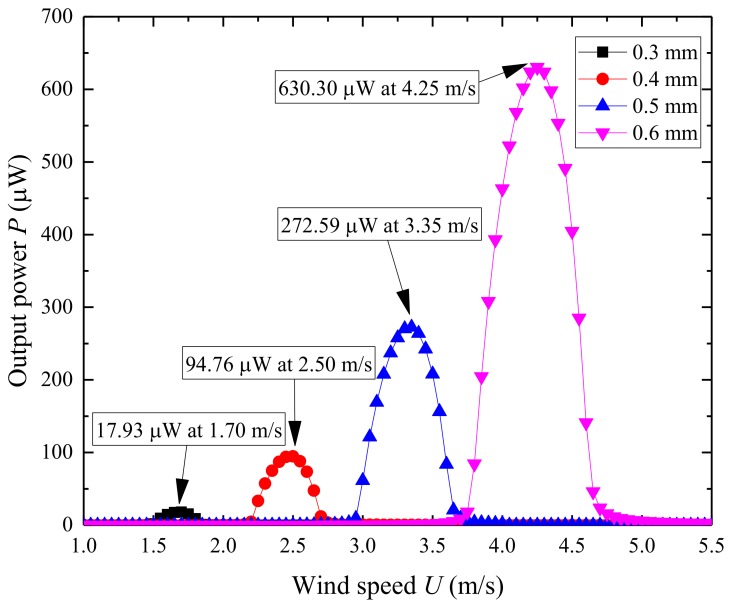
Variation of the output power *P* of UPEH as a function of the thickness of substrate layer.

Figure 7 shows the variation of the first natural frequency of UPEH and electromechanical coupling coefficient as a function of the thickness of the substrate layer. The first natural frequency of the UPEH is increased from 8.28 Hz to 20.79 Hz, and electromechanical coupling coefficient changes from −0.0005 to −0.0016 as the thickness of the substrate layer increases from 0.3 mm to 0.6 mm. A higher first natural frequency results in a larger corresponding wind speed region. As a result, the resonant wind speed is increased from 1.70 m/s to 4.25 m/s, as shown in Figure 8. The maximum output power is also increased from 17.93 µW to 630.29 µW because the lift force on the attached cylinder increases with higher resonant wind speed. The thickness of the substrate also makes an impact on the working bandwidth of wind speed. A greater thickness of the substrate layer results in a wider bandwidth. As a result, the UPEH can get better output power performance with a thicker substrate in the corresponding wind speed region.

**Figure 9:**
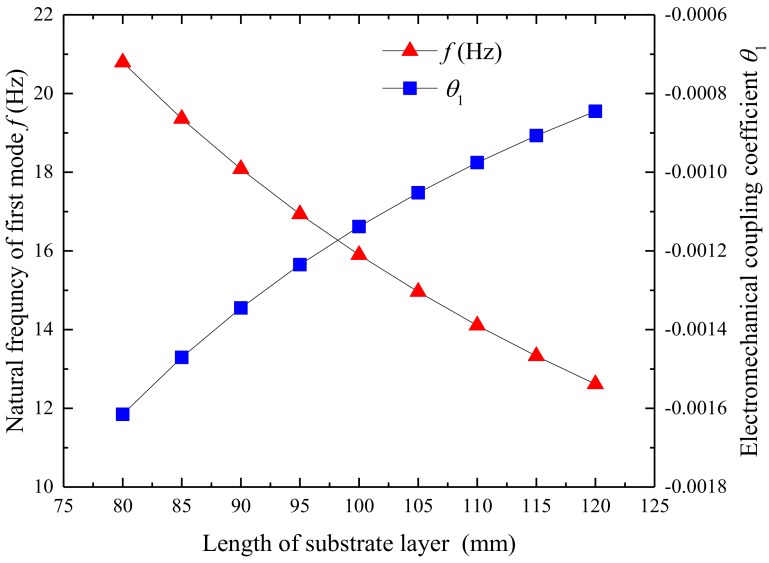
Variation of the first natural frequency and electromechanical coupling coefficient of UPEH as a function of the length of substrate layer.

**Figure 10:**
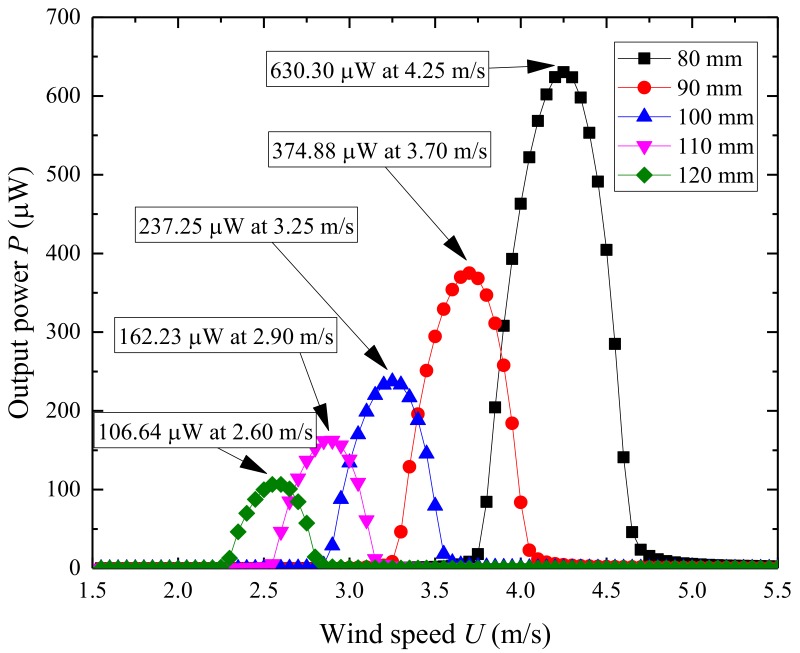
Variation of the output power *P* as a function of the length of substrate layer.

Figure 9 illustrates the changes of the first natural frequency and electromechanical coupling coefficient relating to the length of the substrate layer. Figure 10 illustrates the output power of the UPEH as a function of wind speed. The first natural frequency is 20.79 Hz when the length of the substrate layer is 80 mm, while it is 12.61 Hz when the length of the substrate layer is 120 mm. The electromechanical coupling coefficient changes from −0.0016 to −0.0008. The length of the substrate has little effect on the width of the synchronization region. The maximum output power also decreases from 630.30 µW to 106.64 µW when the length of the substrate layer changes from 80 mm to 120 mm. The main reason for this result is that a longer substrate leads to a lower natural frequency. The results show that an energy harvester with a shorter substrate can provide better output power performance than with a longer substrate within the length range of this study.

**Figure 11:**
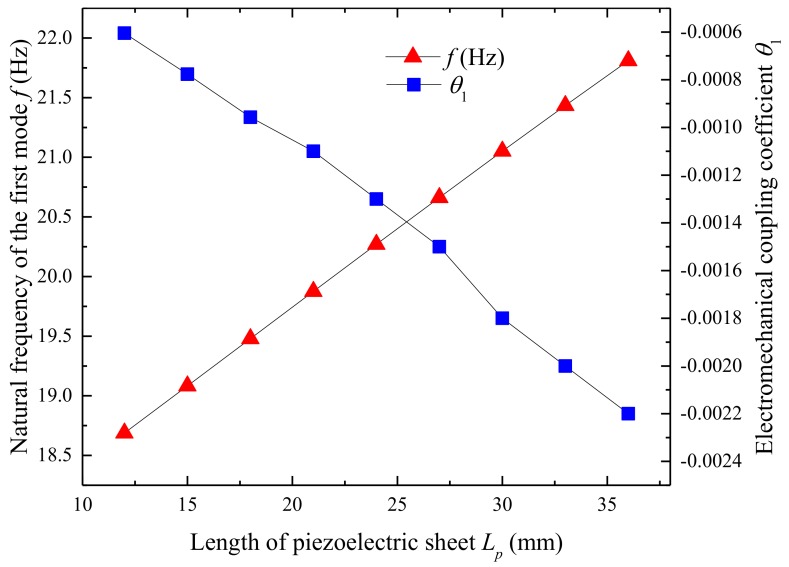
Variation of the first natural frequency and electromechanical coupling coefficient as a function of the length of piezoelectric sheet.

**Figure 12:**
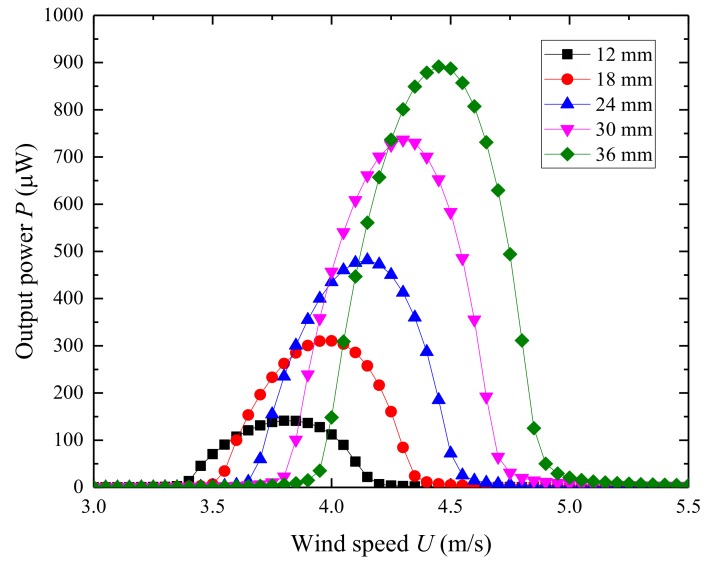
Variation of the output power *P* related to the length of piezoelectric sheet.

Figure 11 shows the effects of the length of the piezoelectric sheet on the natural frequency and electromechanical coupling coefficient. The natural frequency increases from 18.69 Hz to 21.81 Hz and the electromechanical coupling coefficient varies from −0.0006 to −0.0022 as the length of the piezoelectric sheet changes from 12 mm to 36 mm. The results show that the length of the piezoelectric sheet has a distinct influence on the natural frequency of the UPEH. Figure 12 illustrates the output power relating to the length of the piezoelectric sheet. The maximum output power varies from 141.23 µW at a wind speed of 3.80 m/s when the length of the piezoelectric sheet is 12mm to 891.69 µW at a wind speed of 4.45 m/s when the length is 36mm. The main reasons for the results are the higher natural frequency and higher electromechanical coupling coefficient.

**Figure 13:**
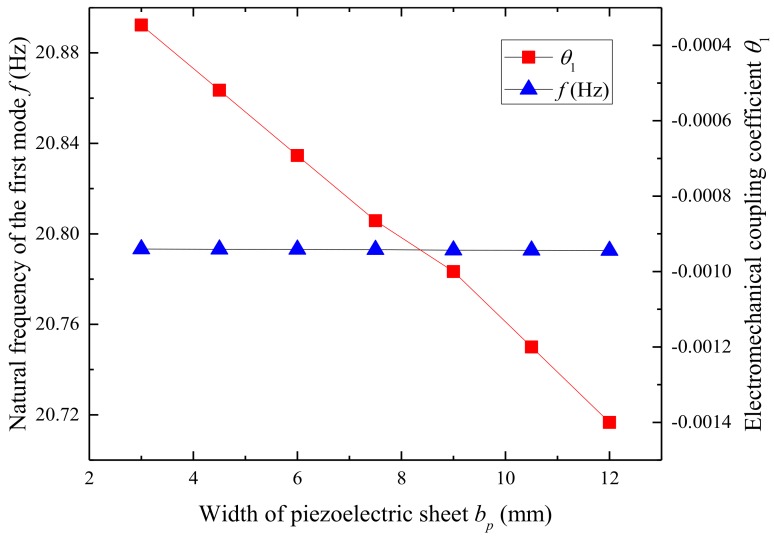
Variation of the first natural frequency and electromechanical coupling coefficient as a function of the width of piezoelectric sheet.

**Figure 14:**
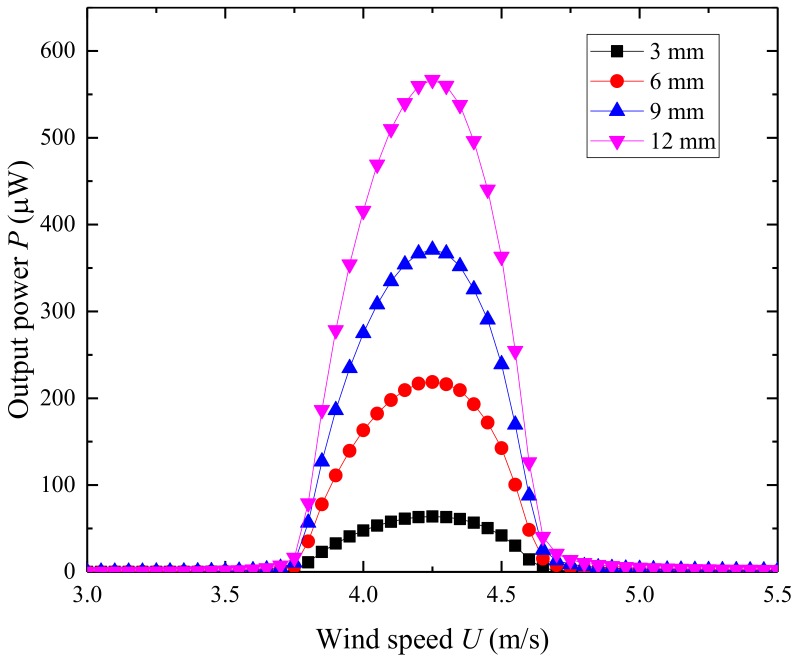
Variation of the output power *P* as a function of the width of piezoelectric sheet.

Figure 13 and Figure 14 show the variation of the first natural frequency, electromechanical coupling coefficient, and output power related to the length of the substrate layer, respectively. Figure 13 illustrates that the width of the piezoelectric sheet has barely any effect on the first natural frequency of the UPEH but has a significant influence on the electromechanical coupling coefficient. The electromechanical coupling coefficient is −0.00035 when the width of the piezoelectric sheet is 3 mm while the electromechanical coupling coefficient is −0.0014 when the width of the piezoelectric sheet is 12 mm. From Figure 14 we can observe the optimum wind speeds for harvesters with different widths of the piezoelectric sheet are almost the same as they have nearly the same natural frequency. However, an energy harvester with a greater width piezoelectric sheet can obtain higher output power performance because it has a larger electromechanical coupling coefficient. 

To compare the efficiency of a UEPH and a TPEH, we need to compare the working volume for the same output power, which means the vibrations of piezoelectric cantilevers for UEPH and TPEH need to be the same. The working volume *V_T_* for the TPEH is [37]

(35) $$V_{T}=2L_{c}\left(\frac{D}{2}+w\left(L_{s}\right)\right)\left(L_{s}+D\right)$$

The working volume *V_U_* for UPEH is

(36) $$V_{U}=2D\left(\frac{D}{2}+w\left(L_{s}\right)\right)\left(L_{s}+L_{c}\right)$$

Then we divide these two values to get the efficiency ratio *r*

(37) $$r=\frac{V_{U}}{V_{T}}=1-\frac{L_{s}}{L_{c}\left(L_{s}+D\right)}\left(L_{c}-D\right)$$

If the length value of cylinder extension is bigger than the diameter value of cylinder, the efficiency ratio *r* is less than 1, which means a UPEH is better than a TPEH from the view point of working space. For the case in experiments study in this paper, the efficiency ratio *r* is 66.7%.

## 5. Conclusions

In this paper, an upright piezoelectric energy harvester (UPEH) composed of a piezoelectric cantilever with an upright cylinder attachment is investigated for converting wind kinetic energy into electricity. The UPEH has the advantages of less working space and ease of setting up an array of multiple energy harvesters over the T-shaped piezoelectric energy harvester (TPEH). Fluid-added mass caused by the moving cylinder is taken into consideration in the theory. The Lagrange’s equation is established to obtain the governing coupled equation through Galerkin discretization. Experiments were designed and conducted. The experimental results show that this upright energy harvester can generate 635.04 μW at a wind speed of 4.20 m/s while the theoretical prediction is 630.29 μW at a wind speed of 4.25 m/s, showing a close match. The comparison appears in a good agreement between the experimental results and the theoretical predictions in the synchronization region. The peak power output is predicted by a theoretical model within 1% error. The theoretical prediction agrees well with the experimental results. The effects of tip mass of cylinder, thickness and width of the substrate layer, and piezoelectric sheet on the first natural frequency, electromechanical coupling coefficient, and performance of the UPEH were further analyzed. The natural frequency of the energy harvester and the synchronization region wind velocity decrease with increasing cylinder mass. However, the width of the synchronization region widens as the mass of the cylinder reduces. Greater thickness of the substrate layer results in a lower natural frequency of harvester and wider bandwidth of the synchronization region. Energy harvesters with a greater width of the piezoelectric sheet can generate higher output power although they have nearly the same natural frequency. An energy harvester with a shorter substrate results in a higher natural frequency and provides better output power performance at higher wind speeds. It is found that a UPEH achieves better performance with a lighter tip mass of cylinder and thicker and shorter substrate at synchronization wind speed. The application value of the UPEH will be applicable to the wireless power supply of sensors or communication systems in remote areas.

## Figures and Tables

**Table 1:** The geometric and physical properties of energy harvesting system.

Parameters	Properties	Values
*ρ_p_*	Density of the piezoelectric layer (kg/m^3^)	5440
*ρ_s_*	Density of the substrate layer (aluminum; kg/m^3^)	2700
*ρ_f_*	Fluid density (air; kg/m^3^)	1.2
*ρ_c_*	Density of the cylinder (foam; kg/m^3^)	27.9
*E_p_*	Young modulus of the piezoelectric layer (Gpa)	30.336
*E_s_*	Young modulus of the substrate layer (Gpa)	69.5
*L_p_*	Length of the piezoelectric layer (mm)	28
*L_c_*	Length of cylinder (mm)	80
*D*	Diameter of cylinder(mm)	40
*L_s_*	Length of the substrate layer (mm)	80
*b_p_*	Width of the beam (mm)	17
*b_1_*	Width of active piezoelectric layer (mm)	14
*h_p_*	Thickness of the piezoelectric layer (mm)	0.3
*h_s_*	Thickness of the substrate layer (mm)	0.65
*e*_31_	Piezoelectric stress coefficient (C/m^2^)	−5.157
*ε*_33_	Piezoelectric dielectric constant (nF/m)	23.556
*d_33_*	Piezoelectric constant (pC/N)	400
*ζ_1_*	Damping ratio of first mode	0.49%

## Appendix A

The state-space forms of the governing equations are denoted as follows:

(A1) $$X_{1}=r,\;X_{2}=\dot{r},\;X_{3}=V,\;X_{4}=q,\;X_{5}=\dot{q}$$

Equation (27) can be written into the state space form as

(A2) $$\begin{array}{l} {\dot{X}}_{1}=\dot{r}\\ {\dot{X}}_{2}=\ddot{r}=-\left(\omega_{1}{}^{2}+\mu_{1}\right)r-(2\zeta_{1}\omega_{1}+\eta_{1})\dot{r}-\theta_{1}V+Kq\\ {\dot{X}}_{3}=\dot{V}=\frac{\theta_{1}}{C_{p}}\dot{r}-\frac{1}{C_{p}R}V\\ {\dot{X}}_{4}=\dot{q}\\ {\dot{X}}_{5}=\ddot{q}=-\varepsilon \omega_{f}(q^{2}-1)\dot{q}-\omega_{f}{}^{2}q+\frac{A}{D}\left(\phi_{12}\left(L_{s}\right)+\frac{L_{c}}{2}{\phi^{\prime }}_{12}\left(L_{s}\right)\right)\left(Kq-\left(\omega_{1}{}^{2}+\mu_{1}\right)r-\theta_{1}V-(2\zeta_{1}\omega_{1}+\eta_{1})\dot{r}\right) \end{array}$$

Then the above equations can be rewritten as

(A3) $$\begin{array}{l} \dot{X}=\\ \left[\begin{array}{c} X_{2}\\ -\left(\omega_{1}{}^{2}+\mu_{1}\right)X_{1}-(2\zeta_{1}\omega_{1}+\eta_{1})X_{2}-\theta_{1}X_{3}+KX_{4}\\ \frac{\theta_{1}}{C_{p}}X_{2}-\frac{1}{C_{p}R}X_{3}\\ X_{5}\\ -\varepsilon \omega_{f}(X_{4}{}^{2}-1)X_{5}-\omega_{f}{}^{2}X_{4}+\frac{A}{D}\left(\phi_{12}\left(L_{s}\right)+\frac{L_{c}}{2}{\phi^{\prime }}_{12}\left(L_{s}\right)\right)\left(KX_{4}-\left(\omega_{1}{}^{2}+\mu_{1}\right)X_{1}-\theta_{1}X_{3}-(2\zeta_{1}\omega_{1}+\eta_{1})X_{2}\right) \end{array}\right] \end{array}$$
